# Short-term BMI-SDS change during child and adolescent psychiatric hospitalization: associations with antipsychotic treatment and length of stay

**DOI:** 10.3389/fpsyt.2026.1942502

**Published:** 2026-10-02

**Authors:** Marjetka Švigelj, Maja Drobnič Radobuljac, Barbara Plemeniti Tololeski, Anja Turin, Primož Kotnik

**Affiliations:** 1 Faculty of Medicine, University of Ljubljana, Ljubljana, Slovenia; 2 Unit for Intensive Child and Adolescent Psychiatry, Center for Mental Health, University Psychiatric Clinic Ljubljana, Ljubljana, Slovenia; 3 Department for Child Psychiatry, University Children’s Hospital Ljubljana, University Medical Centre Ljubljana, Ljubljana, Slovenia; 4 Department of Endocrinology, Diabetes and Metabolism, University Children’s Hospital Ljubljana, Ljubljana, Slovenia

**Keywords:** antipsychotics, BMI-SDS, child and adolescent psychiatry, olanzapine, pediatric psychopharmacology, psychiatric hospitalization, weight gain

## Abstract

Children and adolescents receiving inpatient psychiatric treatment may be at increased risk of short-term weight gain-related changes, particularly during antipsychotic exposure. In this retrospective cohort study we analyzed a patient-level cohort of 776 unique patients younger than 18 years treated between 2022 and 2024, after exclusion of eating disorder diagnoses and repeated hospitalizations. Clinical data were extracted from electronic medical records and included demographic and clinical characteristics, antipsychotic treatment, and anthropometric measurements. The median hospitalization duration was 3.0 days (IQR 2.0-7.0). Repeated BMI-SDS measurements permitting calculation of BMI-SDS change were available for 228 patients. Mean BMI-SDS change was +0.071 (SD = 0.242). BMI-SDS increased significantly among patients exposed to antipsychotics (n = 152; mean change +0.107, p < 0.001), whereas no significant change was observed among patients without antipsychotic exposure (n = 76). BMI-SDS change differed across antipsychotic treatment groups (Kruskal-Wallis p = 0.0006; ϵ² = 0.081). However, olanzapine monotherapy was not significantly different from no antipsychotic treatment after correction for multiple comparisons, although exploratory analysis showed a higher risk of BMI-SDS increase ≥0.5 with olanzapine exposure (NNH = 3.7, 95% CI 2.2–10.2). In multivariable linear regression analysis, longer hospitalization (B = 0.0035 per day, 95% CI 0.002-0.005, p < 0.001) and any antipsychotic exposure (B = 0.0668, 95% CI 0.003-0.131, p = 0.042) were associated with higher follow-up BMI-SDS after adjustment for baseline BMI-SDS. Sex, age and F20-F29 diagnosis were not significantly associated with follow-up BMI-SDS. These findings suggest that short-term BMI-SDS change during child and adolescent psychiatric hospitalization is associated with antipsychotic treatment and length of hospitalization. The results do not support a significant olanzapine dose-response relationship and should be interpreted as anthropometric associations rather than evidence of metabolic dysfunction or causality.

## 1 Introduction

Childhood and adolescent obesity is an important public health concern because excess weight in youth is associated with long-term cardiovascular, endocrine, and psychosocial consequences (1, 2). Young people with psychiatric disorders may be especially vulnerable to weight gain due to a combination of illness-related behavioral factors, reduced physical activity, disrupted eating patterns, and adverse metabolic effects of psychopharmacological treatment (2, 3).

Second-generation antipsychotics (e.g., risperidone, aripiprazole, quetiapine, and olanzapine) are widely used in child and adolescent psychiatry not only for psychotic disorders, but also for mood disorders, severe emotional and behavioral dysregulation, agitation, and other complex clinical presentations (5). Although these medications may be clinically effective, many are associated with clinically relevant adverse effects, including increased appetite, rapid weight gain, dyslipidemia, and insulin resistance (4, 5). Among them, olanzapine is consistently recognized as one of the agents with the highest risk for weight gain and metabolic disturbance (6, 7).

Early weight gain during psychiatric treatment is clinically important because it may predict persistent obesity and future cardiometabolic morbidity. In children and adolescents, even modest short-term increases in weight may be relevant, particularly when they occur during sensitive developmental periods and in already vulnerable patient groups. Identifying factors associated with weight gain in routine psychiatric care could therefore improve risk stratification, inform prescribing decisions, and support the early implementation of preventive interventions (3, 6).

The present study complements existing prospective pediatric evidence (8) by examining BMI-SDS change and antipsychotic treatment patterns, including polypharmacy, in routine psychiatric inpatient care. The primary aim of this study was to examine short-term BMI-SDS change during psychiatric hospitalization in children and adolescents according to antipsychotic treatment group. Secondary analyses examined associations with individual antipsychotics, including olanzapine, and diagnostic subgroup. Exploratory analyses investigated clinical predictors of BMI-SDS increase and compared changes in BMI-SDS and weight-SDS.

## 2 Materials and methods

### 2.1 Study design and setting

This retrospective observational study was conducted using routinely collected clinical data from children and adolescents hospitalized between 2022 and 2024 at the Unit for Intensive Child and Adolescent Psychiatry of the University Psychiatric Clinic in Ljubljana, the only intensive psychiatric facility for children and adolescents in Slovenia. The study was approved by The National Medical Ethics Committee of the Republic of Slovenia (application number 0120-633/2025-2711-8).

### 2.2 Participants

Inclusion criteria were age <18 years and hospitalization at the study unit between January 1, 2022, and December 31, 2024. Exclusion criteria were a diagnosis of an eating disorder (F50.0-F50.9). No *a priori* sample-size calculation was performed; all patients meeting the eligibility criteria during the predefined study period were included. For patients with repeated hospitalizations during the study period, only the first eligible hospitalization was retained. Cohort selection is shown in **Figure 1**.

**Figure 1:**
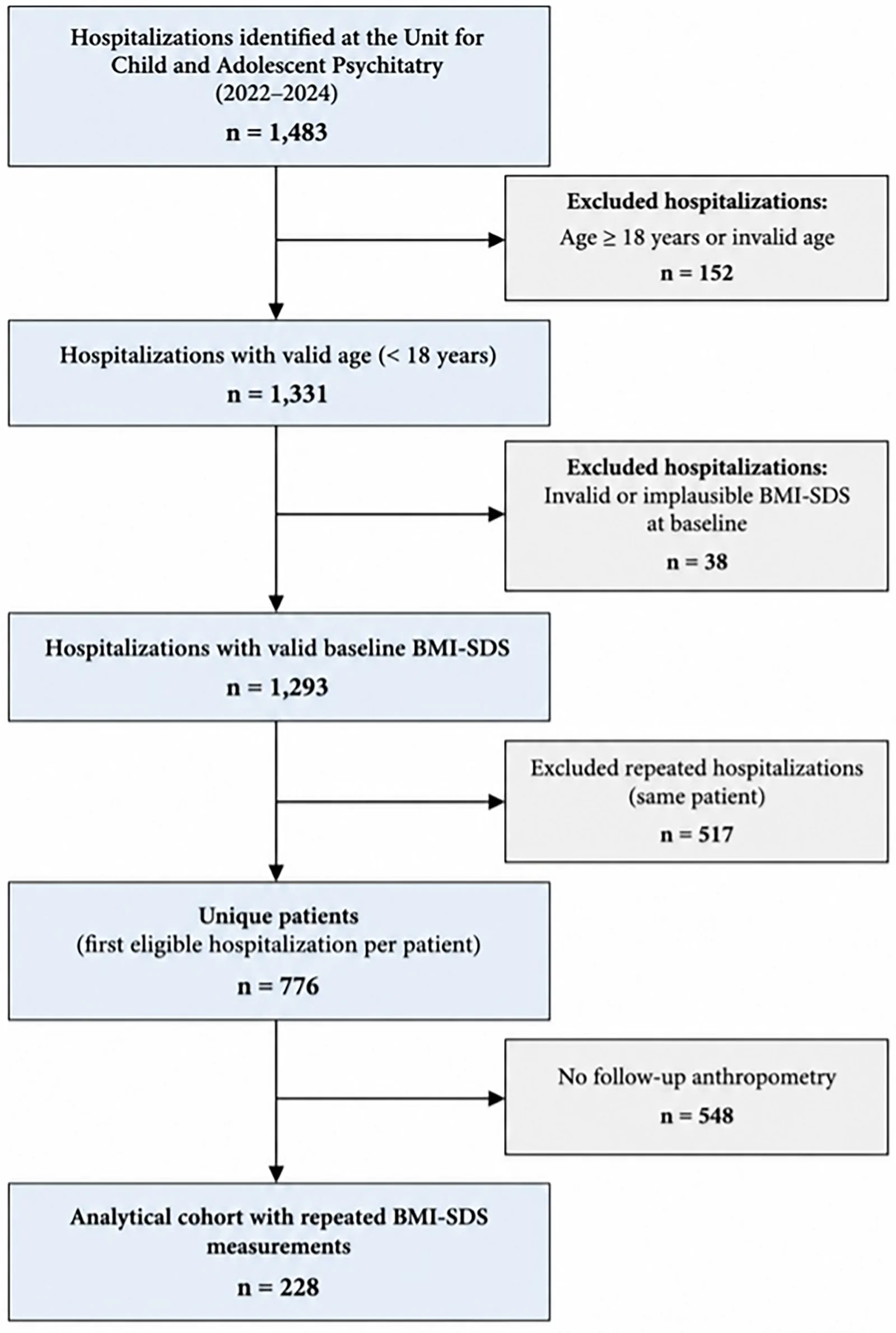
Flowchart of cohort selection.

The analysis was conducted at the patient level, with each patient contributing only one hospitalization to the analytical cohort. This approach was used to reduce potential bias and avoid clustering arising from repeated hospitalizations of the same patient.

### 2.3 Data collection

Clinical data were extracted from electronic medical records. The following variables were recorded: age at admission, sex, duration of hospitalization, psychiatric diagnoses at discharge, BMI-SDS at the first available anthropometric assessment during hospitalization, BMI-SDS at the last available anthropometric assessment during hospitalization, antipsychotic exposure, and antipsychotic dosage, including calculated mean daily dose. BMI standard deviation scores (BMI-SDS) were calculated according to the World Health Organization (WHO) growth reference for school-aged children and adolescents (5–19 years), adjusted for age and sex (9). Anthropometric measurements, including body weight and height, were routinely performed by nursing staff during hospitalization, preferably in the morning, in a fasting state, and in underwear or light clothing whenever feasible. Information on equipment calibration, detailed height-measurement procedures, and hydration status was not systematically available. Antipsychotic exposure was defined as documented treatment with an antipsychotic medication during hospitalization. For olanzapine, quetiapine, clozapine, risperidone, aripiprazole, haloperidol, promazine, and sulpiride, the mean daily dose during hospitalization was calculated by dividing the total administered dose by the total number of hospitalization days. Diagnoses were coded according to the International Classification of Diseases, 10th Revision (ICD-10), as recorded in the medical record at discharge, with up to four diagnoses recorded for each patient’s retained hospitalization. BMI-SDS change was defined as the difference between the last and first recorded BMI-SDS values during the retained hospitalization. Analyses of BMI-SDS change were limited to patients with at least two available anthropometric assessments permitting BMI-SDS calculation.

### 2.4 Diagnostic grouping

In addition to descriptive analysis of the most frequent diagnoses, diagnoses were grouped for selected analyses into F20-F29 (schizophrenia, schizotypal and delusional disorders) and other psychiatric diagnoses. The F20-F29 category was examined separately because it represents schizophrenia-spectrum disorders, for which antipsychotic treatment constitutes a central component of pharmacological management.

### 2.5 Statistical analysis

Descriptive statistics were used to summarize demographic and clinical characteristics. Continuous variables are presented as means and standard deviations or medians and interquartile ranges (IQR), as appropriate; categorical variables are presented as counts and percentages. Data were screened for implausible values before analysis. One clearly implausible BMI-SDS change of 3.5 units during a 3-day hospitalization was identified and excluded from the analytical dataset. The distribution of continuous variables was assessed using the Shapiro–Wilk test. Parametric or non-parametric statistical tests were applied as appropriate based on the distribution of the data.

Associations between mean daily olanzapine dose and BMI-SDS change were examined using Spearman’s rank correlation among olanzapine-exposed patients with available BMI-SDS change data. Patients not exposed to olanzapine were excluded from the dose-response analysis and were not coded as having a dose of zero. Correlations were calculated for hospitalization duration and daily doses of individual antipsychotic medications, including olanzapine, quetiapine, clozapine, risperidone, aripiprazole, haloperidol, promazine and sulpiride. Drug-specific correlation analyses were considered exploratory and were not adjusted for multiple testing.

Changes in BMI-SDS between the first and follow-up measurements were assessed using paired t-tests and Wilcoxon signed-rank tests, separately for patients with and without antipsychotic exposure. BMI-SDS change between patients with and without antipsychotic exposure was compared using the Mann-Whitney U test. Differences in BMI-SDS change across antipsychotic treatment groups were assessed using Kruskal-Wallis test. Dunn’s *post-hoc* pairwise comparisons with Holm correction for multiple testing were performed where appropriate, and epsilon-squared was reported as the overall effect size. For group comparisons, antipsychotic exposure was categorized as no antipsychotic treatment, individual-agent monotherapy, polypharmacy including olanzapine, or polypharmacy without olanzapine.

A multivariable linear regression analysis was performed with follow-up BMI-SDS as the dependent variable and baseline BMI-SDS, age at admission, sex, hospitalization duration, any antipsychotic exposure, and F20-F29 diagnosis as independent variables. Sex was coded as male versus female (reference), any antipsychotic exposure as yes versus no (reference), and F20–F29 diagnosis as present versus absent (reference). Follow-up BMI-SDS rather than BMI-SDS change was modeled to account for baseline BMI-SDS while avoiding mathematical coupling between baseline values and change scores. Model assumptions were evaluated using residual diagnostics, and multicollinearity was assessed using variance inflation factors (VIFs).

Statistical significance was set at p < 0.05. Analyses were performed in Jupyter Notebook environment, using Python 3.10.18, NumPy 2.2.6, pandas 2.3.3, SciPy 1.15.3, scikit-learn 1.7.2, statsmodels 0.15.0, scikit-posthocs 0.16.1 and tableone 0.9.6.

## 3 Results

### 3.1 Sample characteristics

The demographic and clinical characteristics of the study cohort are summarized in **Table 1**. A total of 776 unique patients met the eligibility criteria and were included in the patient-level cohort (**Figure 1**). Of these, 228 patients (29.4%) had repeated BMI-SDS measurements available for analysis, while 548 (70.6%) did not have an available follow-up BMI-SDS measurement and were therefore not included in analyses requiring BMI-SDS change or follow-up BMI-SDS. Among the 228 patients with repeated measurements, mean BMI-SDS change was +0.071 (SD = 0.242; range -0.501 to 1.153).

**Table 1:** Characteristics of the study population according to antipsychotic drug use.

Characteristic	Overall (n = 776)	No drug (n = 321)	Drug use (n = 455)	P-value
**Sex, n (%)**				**0.006**
Female	532 (68.6)	238 (74.1)	294 (64.6)	
Male	244 (31.4)	83 (25.9)	161 (35.4)	
**Age at admission years, median [Q1, Q3]**	16.0 [14.3, 17.0]	15.4 [14.2, 16.7]	16.1 [14.5, 17.1]	**0.003**
**Length of hospitalization, days, median [Q1, Q3]**	3.0 [2.0, 7.0]	3.0 [1.0, 5.0]	4.0 [2.0, 7.5]	**<0.001**
**BMI change, median [Q1, Q3]**	0.0 [0.0, 0.0]	0.0 [0.0, 0.0]	0.0 [0.0, 0.0]	**0.013**
**Schizophrenia spectrum diagnosis (F20–F29), n (%)**				**<0.001**
No	720 (92.8)	314 (97.8)	406 (89.2)	
Yes	56 (7.2)	7 (2.2)	49 (10.8)	
**Drug group, n (%)**				**<0.001**
None	321 (41.4)	321 (100.0)	0 (0.0)	
Aripiprazole	36 (4.6)	0 (0.0)	36 (7.9)	
Haloperidol	5 (0.6)	0 (0.0)	5 (1.1)	
Olanzapine	35 (4.5)	0 (0.0)	35 (7.7)	
Polypharmacy (without olanzapine)	33 (4.3)	0 (0.0)	33 (7.3)	
Polypharmacy (with olanzapine)	26 (3.4)	0 (0.0)	26 (5.7)	
Promazine	2 (0.3)	0 (0.0)	2 (0.4)	
Quetiapine	98 (12.6)	0 (0.0)	98 (21.5)	
Risperidone	211 (27.2)	0 (0.0)	211 (46.4)	
Sulpiride	9 (1.2)	0 (0.0)	9 (2.0)	
**Weight-SDS change, median [Q1, Q3]^a^**	0.0 [−0.1, 0.1]	−0.0 [−0.1, 0.1]	0.1 [−0.0, 0.2]	**0.002**
**BMI-SDS change, median [Q1, Q3]^a^**	0.0 [−0.1, 0.2]	−0.0 [−0.1, 0.1]	0.1 [−0.1, 0.2]	**0.003**

Values are presented as *n* (%) or median [Q1, Q3], as appropriate.

*P*-values compare patients with versus without antipsychotic drug exposure. Statistically significant *p*-values (<0.05) are shown in bold.

^a^SDS-weight and SDS-BMI change were available for 228 of 776 observations (548 missing).

### 3.2 Antipsychotic exposure and BMI-SDS change

Among the 228 with repeated BMI-SDS measurements, 152 were exposed to antipsychotic medication and 76 were not. In antipsychotic-exposed patients, mean BMI-SDS increased from 0.331 to 0.438, corresponding to a mean change of +0.107 (paired t-test p < 0.0001). In patients without antipsychotic exposure, mean BMI-SDS remained unchanged from 0.455 to 0.455, corresponding to a mean change of +0.001; (paired t-test p = 0.968; **Supplementary Figure S2**). BMI-SDS change was significantly greater among patients exposed to antipsychotics than among those without antipsychotic exposure (Mann–Whitney U test, p = 0.017; **Supplementary Figure S2**). Overall, 13 of 228 patients (5.7%) had a BMI-SDS increase ≥ 0.5 during hospitalization.

### 3.3 Differences between antipsychotic treatment groups

BMI-SDS change differed significantly across antipsychotic treatment groups (Kruskal–Wallis H = 23.598, p = 0.0006, ϵ² = 0.081; n = 225; **Figure 2**). Median BMI-SDS change was −0.007 (IQR −0.125 to 0.123) in patients without antipsychotic exposure and +0.193 (IQR 0.000 to 0.388) in patients receiving olanzapine monotherapy. However, this difference was not statistically significant after correction for multiple comparisons (Dunn’s *post-hoc* test with Holm correction, p = 0.1541). Significant pairwise differences were observed between patients without antipsychotic exposure and the polypharmacy group without olanzapine (p = 0.024), and between patients without antipsychotic exposure and the polypharmacy group including olanzapine (p = 0.045). No significant pairwise differences were observed between individual antipsychotic monotherapy groups after Holm correction. Among the 27 olanzapine-exposed patients with available BMI-SDS change data, mean daily olanzapine dose was not significantly associated with BMI-SDS change (Spearman’s ρ = −0.213, p = 0.286). In an exploratory threshold analysis, a BMI-SDS increase ≥0.5 occurred in 29.6% of olanzapine-exposed patients compared with 2.5% of patients not exposed to olanzapine, corresponding to a number needed to harm (NNH) of 3.7 (95% CI 2.2–10.2; Fisher’s exact p < 0.0001). BMI-SDS change across tertiles of mean daily olanzapine dose is shown in **Supplementary Figure S1**.

**Figure 2:**
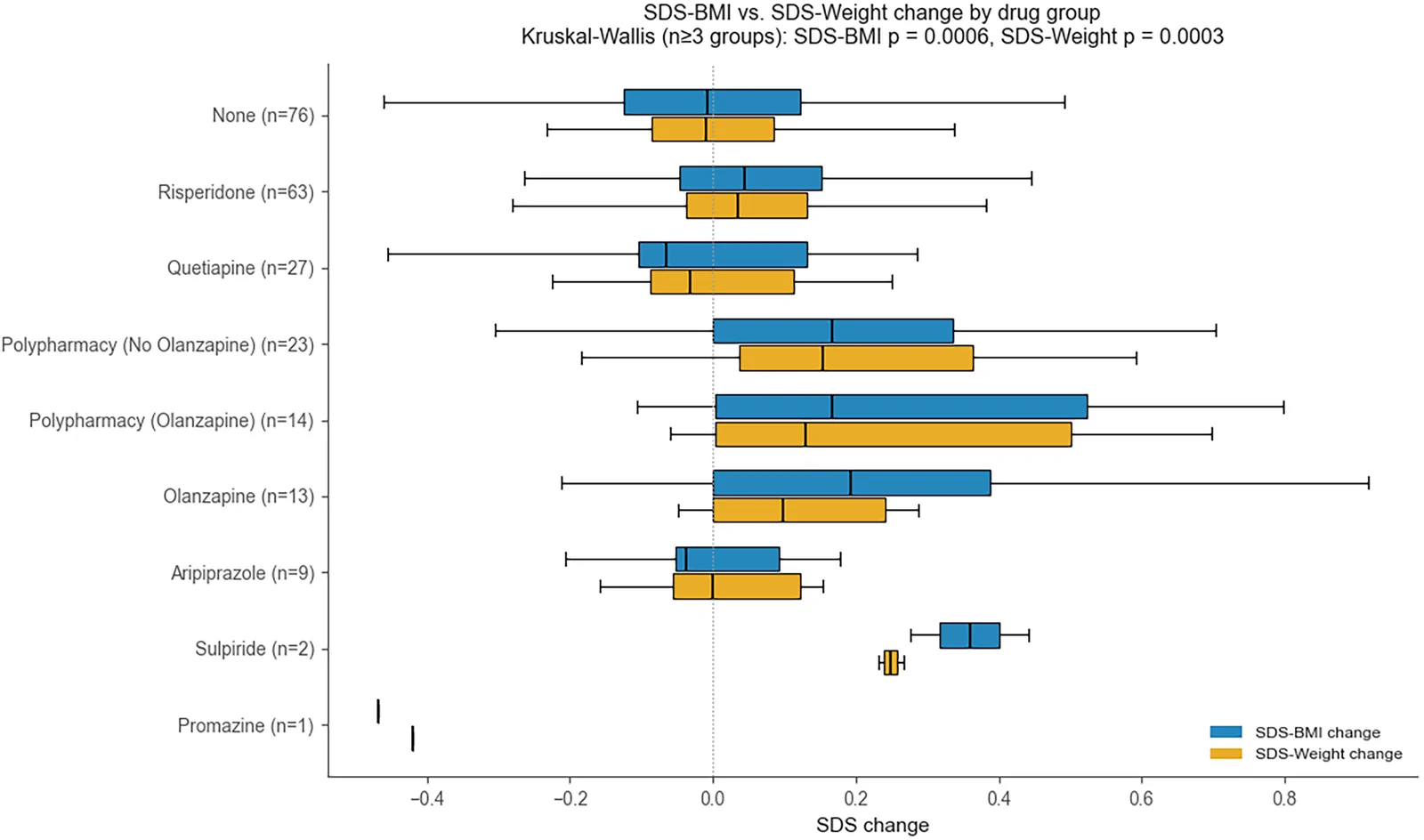
BMI-SDS vs. Weight-SDS change by drug group.

### 3.4 Multivariable analysis of follow-up BMI-SDS

The multivariable linear regression model included 228 patients. The model was statistically significant (F(6,221)=1267, p < 0.001) and explained 97.2% of the variance in follow-up BMI-SDS (R² = 0.972; adjusted R² = 0.971). The intercept was −0.0182 (SE = 0.162, 95% CI −0.338 to 0.302, p = 0.911). Baseline BMI-SDS was strongly associated with follow-up BMI-SDS (B = 0.9521, SE = 0.011, 95% CI 0.930–0.974, p < 0.001). Longer hospitalization was associated with higher follow-up BMI-SDS (B = 0.0035 per day, SE = 0.001, 95% CI 0.002–0.005, p < 0.001), as was any antipsychotic exposure (B = 0.0668, SE = 0.033, 95% CI 0.003–0.131, p = 0.042) (See **Figure 3**). Age (B = −0.0015, SE = 0.010, 95% CI −0.022 to 0.019, p = 0.883), sex (B = 0.0376, SE = 0.033, 95% CI −0.028 to 0.103, p = 0.260), and F20–F29 diagnosis (B = 0.0820, SE = 0.047, 95% CI −0.011 to 0.175, p = 0.085) were not significantly associated with follow-up BMI-SDS. Multicollinearity was minimal, with VIF values ranging from 1.02 to 1.14 across predictors.

**Figure 3:**
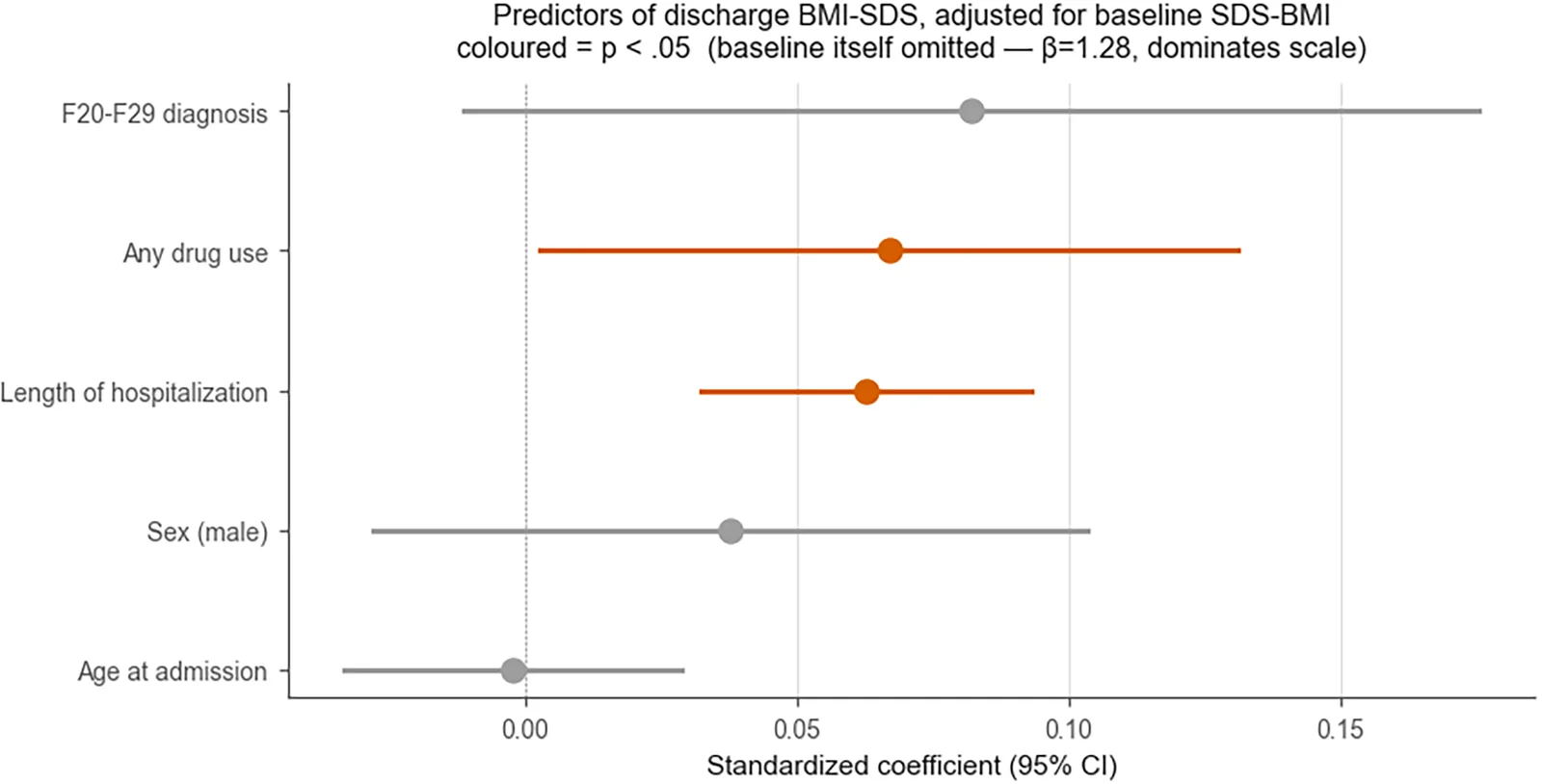
Multivariable associations with follow-up BMI-SDS.

## 4 Discussion

This retrospective study examined short-term BMI-SDS change during psychiatric hospitalization in children and adolescents in a real-world inpatient setting. The main findings indicate that BMI-SDS increased modestly among patients with repeated anthropometric measurements and that this increase was more pronounced among patients exposed to antipsychotic medication. In the multivariable model, longer hospitalization duration (B = 0.0035 per day, 95% CI 0.002–0.005) and any antipsychotic exposure (B = 0.0668, 95% CI 0.003–0.131) were associated with higher follow-up BMI-SDS after adjustment for baseline BMI-SDS and other available covariates. Although BMI-SDS change differed across antipsychotic treatment groups, olanzapine monotherapy was not significantly different from no antipsychotic treatment after correction for multiple comparisons, and no significant association between mean daily olanzapine dose and BMI-SDS change was observed.

The overall mean BMI-SDS change was modest (+0.071), and its clinical relevance should therefore be interpreted cautiously. Among patients with repeated measurements, 13 of 228 (5.7%) had a BMI-SDS increase of at least 0.5. Patients exposed to antipsychotics showed a significant increase in BMI-SDS, whereas no significant change was observed among patients without antipsychotic exposure. These findings are consistent with previous evidence that antipsychotic treatment in children and adolescents may be accompanied by early weight-related changes (10). Previous pediatric studies have demonstrated substantial early weight and metabolic changes following initiation of second-generation antipsychotics, with clinically relevant differences between individual agents (8, 11). Recent longitudinal evidence further suggests that cardiometabolic risk following antipsychotic initiation may be proportionally greater in children and adolescents than in young adults (12). Our findings are consistent with the broader evidence for early antipsychotic-associated weight change in young patients, although the present study did not demonstrate a significant olanzapine-specific or dose-response effect. However, BMI-SDS is an anthropometric measure and should not be interpreted as direct evidence of metabolic dysfunction or cardiometabolic deterioration.

An important consideration is that measurable BMI-SDS change was observed within the period of inpatient care. However, the exact interval between the first and last anthropometric measurements was not available retrospectively, and total hospitalization duration cannot be assumed to represent the anthropometric observation interval. Consequently, the present data do not allow the precise rate of BMI-SDS change to be established. Over short intervals, changes in measured body weight may reflect not only adipose tissue accumulation but also changes in hydration, glycogen and associated water stores, gastrointestinal contents, food intake, physical activity, clothing, and ordinary measurement variability. Potential neuroendocrine, appetite-related, or insulin-mediated effects of antipsychotic treatment are biologically plausible (6, 7), but these mechanisms were not measured in the present study and should therefore be considered hypotheses rather than explanations established by the current data. This distinction is particularly important when interpreting early anthropometric changes in an inpatient setting.

Hospitalization duration was associated with higher follow-up BMI-SDS after adjustment for baseline BMI-SDS and the other included covariates. Longer hospitalization may reflect greater opportunity for anthropometric change, longer medication exposure, greater illness severity, treatment complexity, reduced physical activity, changes in usual routines, or inpatient environmental factors related to food intake and movement (13). Neither the exact interval between anthropometric measurements nor the duration of medication exposure was available. Because duration of stay is unlikely to be a purely independent biological factor, it should be interpreted as a composite marker of observation opportunity, treatment intensity and clinical complexity rather than a direct cause of BMI-SDS change. In this setting, many admissions are relatively short and focused on acute stabilization, which suggests that hospitalization duration may partly reflect greater opportunity for treatment exposure and anthropometric change rather than a direct biological effect.

Olanzapine was associated with a numerically greater BMI-SDS increase, although olanzapine monotherapy was not significantly different from no antipsychotic treatment after correction for multiple comparisons. In addition, higher mean daily olanzapine dose was not significantly associated with greater BMI-SDS change. These findings should be interpreted alongside the well-established weight-gain risk profile of olanzapine in pediatric populations (6, 7), but do not demonstrate a medication-specific or dose-dependent effect of olanzapine in the present study. Several biological mechanisms may contribute to olanzapine-associated weight gain. Olanzapine has strong antagonistic effects on histaminergic, serotonergic, and muscarinic receptors, which are thought to increase appetite, alter satiety regulation, and reduce energy expenditure (14). Evidence also suggests effects on insulin sensitivity and peripheral metabolic pathways that may contribute to weight gain, particularly in younger patients (7). However, these mechanisms were not assessed in the present study and should be considered possible explanations rather than mechanisms demonstrated by our findings.

Notably, BMI-SDS change differed significantly across antipsychotic treatment groups, although no significant pairwise differences were observed between individual antipsychotic monotherapy groups after correction for multiple comparisons. This does not necessarily imply the absence of weight-gain risk for individual agents. Rather, the findings may reflect lower exposure frequency, lower dosing, shorter duration of treatment, heterogeneity of prescribing patterns, or insufficient statistical power for medication-specific analyses (7).

Patients with F20–F29 diagnoses did not show a significantly higher follow-up BMI-SDS after adjustment for baseline BMI-SDS and other covariates. This does not necessarily imply the absence of an association with diagnosis, as the F20-F29 group represented a relatively small proportion of the cohort and the comparison group included a heterogeneous range of psychiatric diagnoses. Furthermore, diagnosis is closely related to treatment patterns and clinical characteristics, which may contribute to confounding by indication.

This study has several limitations. First, the retrospective design limits causal inference. Second, BMI-SDS change could only be assessed in the subset of patients with at least two anthropometric measurements (228 of 776 patients, 29.4%), which may introduce selection bias. Third, anthropometric measurements were obtained as part of routine clinical care rather than according to a standardized research protocol; therefore the exact timing and measurement conditions could not be standardized retrospectively, and the exact interval between the first and follow-up measurements was not available. Furthermore, waist circumference, body-composition measures, blood pressure, and biochemical markers of metabolic function were not systematically available; therefore, the present findings cannot be interpreted as evidence of metabolic dysfunction or cardiometabolic deterioration. Fourth, the dataset did not include potentially relevant covariates such as diet, physical activity, pubertal stage, socioeconomic factors, illness severity, relevant medical and endocrine comorbidities, concomitant medications affecting body weight, prior psychotropic exposure, or precise duration of medication treatment. Fifth, precise information regarding medication initiation and discontinuation, switching, and cumulative exposure was not available. For patients with repeated hospitalizations, only the first eligible hospitalization was retained, and the present study therefore used a patient-level design with one hospitalization per patient. Finally, the single-center tertiary setting, predominantly female cohort, and exclusion of patients with eating disorders may limit the generalizability of the findings to other inpatient psychiatric settings and patient populations.

Despite these limitations, the study has important clinical strengths. It reflects routine practice in a tertiary child and adolescent psychiatric inpatient setting and uses BMI-SDS, which is more appropriate than raw BMI when evaluating weight-related change in a pediatric population. The findings also have practical implications for monitoring. Children and adolescents receiving antipsychotic treatment should undergo structured anthropometric and metabolic monitoring in accordance with pediatric recommendations, including assessment at baseline and during follow-up (15). Where clinically appropriate, structured monitoring may be accompanied by lifestyle counseling and consideration of switching to an antipsychotic with a lower weight-gain liability in patients experiencing antipsychotic-associated weight gain (16). Multidisciplinary approaches to metabolic risk management have also been described in adult psychiatric populations (17, 18); however, this evidence may inform implementation considerations but should not be directly extrapolated to pediatric risk or treatment effects.

In conclusion, BMI-SDS in children and adolescents increased modestly among patients with repeated anthropometric measurements during psychiatric hospitalization. In this retrospective patient-level cohort, longer hospitalization and antipsychotic exposure were associated with higher follow-up BMI-SDS after adjustment for baseline BMI-SDS and other available covariates. Although BMI-SDS change differed across antipsychotic treatment groups, olanzapine monotherapy was not significantly different from no antipsychotic treatment after correction for multiple comparisons, and no significant olanzapine dose-response relationship was observed. These findings support the importance of routine anthropometric monitoring in young psychiatric inpatients receiving antipsychotic treatment but should be interpreted cautiously. Further prospective studies with standardized anthropometric measurements and precisely defined medication exposure are needed to determine the clinical significance and longer-term trajectory of these early changes.

## Data Availability

The raw data supporting the conclusions of this article will be made available by the authors, without undue reservation.

## Supplementary material

Supplementary Figure 1 BMI-SDS change according to tertiles of mean daily olanzapine dose among olanzapine-exposed patients.

Supplementary Figure 2 BMI-SDS and weight-SDS at admission and follow-up according to antipsychotic exposure.
